# Biosynthesized Silver Nanoparticles Using *Morus alba* (White Mulberry) Leaf Extract as Potential Antibacterial and Anticancer Agents

**DOI:** 10.3390/molecules28031213

**Published:** 2023-01-26

**Authors:** Tipaporn Kumkoon, Monrudee Srisaisap, Panadda Boonserm

**Affiliations:** Institute of Molecular Biosciences, Mahidol University, Nakhon Pathom 73170, Thailand

**Keywords:** *Morus alba* extract, silver nanoparticles, antibacterial activity, anticancer activity

## Abstract

In this study, we report the green synthesis of silver nanoparticles (AgNPs) from *Morus alba* or white mulberry leaf extract (MLE) and assess their antibacterial and anticancer potential. The GC–MS analysis of MLE confirmed the existence of phenolic compounds, serving as reducing, capping, and stabilizing agents in the biosynthesis of AgNPs. The MLE-AgNPs were spherical, with an average particle size of 20–44.5 nm and a face-centered cubic structure. EDX spectra confirmed the formation of AgNPs, and a negative zeta potential value (−14.5 mV) suggested their physicochemical stability. Excellent antibacterial activity was demonstrated by MLE-AgNPs against *Acinetobacter baumannii* strains with a MIC of 2 μg/mL, while good activity was observed against other Gram-negative (*Escherichia coli* and *Salmonella typhimurium*) and Gram-positive (*Bacillus subtilis* and *Staphylococcus aureus*) bacteria with a MIC of 32 μg/mL. In vitro cytotoxic effects on MCF-7 (human breast cancer cells) and MCF-10A (normal human mammary epithelial cells) were investigated by the MTT assay. The half-maximal inhibitory concentrations (IC_50_) against MCF-7 cells were 18 and 33 μg/mL for MLE-AgNPs and MLE, respectively, with no effect on normal MCF-10A cells. Altogether, the results support the high antibacterial and anticancer potential of biosynthesized AgNPs by white mulberry leaf extract.

## 1. Introduction

In recent years, there has been a growing interest in using nanoparticles for various biomedical applications, e.g., in targeted drug delivery, bioimaging, biosensors and anticancer therapy [1]. Silver (Ag) is the metal most often used in nanoparticle preparation due to its antibacterial, antiviral, antifungal, antioxidant and anticancer properties [2]. Silver nanoparticles (AgNPs) can be typically prepared using chemical or physical approaches. However, these techniques involve hazardous reagents, time-consuming procedures and specialized equipment. In contrast, biological or green synthesis can overcome these difficulties and is now established as an alternative approach. In green synthesis, natural extracts from various organisms, e.g., plants, algae, fungi or other microorganisms, serve as reducing and capping agents [3,4,5]. The use of plant extracts for AgNP synthesis is preferable to microbe-mediated synthesis due to their eco-friendliness, non-pathogenicity, scalability, and lower cost [3]. Reduction and stabilization of AgNPs are performed by biomolecules in the plant extracts, such as proteins, amino acids, enzymes, polysaccharides, alkaloids, tannins, phenolics, saponins, terpenoids and vitamins [6]. Several plant extracts have been successfully used for AgNP preparation due to their excellent properties for biomedical applications. Mulberry (*Morus* spp., *Moraceae*) is a well-known medicinal plant used to treat stress, hyperlipidemia, hypertension and hyperglycemia [7]. *Morus* spp. is a rich source of phenolic compounds with antioxidant properties, such as flavonoids and anthocyanins [8]. Within *Morus* spp., *Morus alba* (*M. alba*), commonly known as white mulberry, is widely distributed in tropical countries such as China, India, Indonesia, and Thailand, where it is cultivated for its fruits, leaves, bark and roots that provide health benefits [9]. Bioactive compounds extracted from white mulberry exhibited antibacterial activity [10], in vitro cytotoxicity [11], in vivo antioxidant activity [12], in vitro antiproliferative activity against human colon and breast cancer cells [13] and growth inhibition of breast cancer cells in mice [14]. These findings indicate that *M. alba* extracts may potentially eliminate pathogenic microorganisms, and prevent and treat cancer [15].

The present study aims to synthesize AgNPs using the reducing, stabilizing, and capping agents contained in an aqueous leaf extract of *M. alba* (MLE). The AgNPs mediated by MLE (MLE-AgNPs) were analyzed by using UV–Visible spectroscopy, dynamic light scattering (DLS), field emission scanning electron microscopy (FE-SEM) with energy dispersive x-ray spectroscopy (EDX), Fourier-transform infrared spectroscopy (FTIR) and x-ray diffractometry (XRD). The MLE-AgNPs were effective against both Gram-positive and Gram-negative bacteria. The anticancer potential of these MLE-AgNPs was tested against breast cancer cells (MCF-7) and compared with normal human mammary epithelial cells (MCF-10A). We provide evidence that MLE and MLE-AgNPs inhibit the proliferation of MCF-7 breast cancer cells without affecting normal cells (MCF-10A), which may indicate anticancer specific.

## 2. Results and Discussion

### 2.1. Gas Chromatography and Mass Spectrometry (GC–MS) Analysis of an Aqueous Morus alba Leaf Extract (MLE) Revealed the Presence of Significant Bioactive Compounds

An aqueous *M. alba* leaf extract (MLE) was analyzed using GC–MS to identify bioactive compounds that may have acted as a reducing agent for free metal cations (Ag^+^ to Ag^0^), a stabilizing agent of AgNPs during the nucleation phase, and a capping agent for fully grown or stabilized AgNPs (Table 1 and Figure S1 (Supplementary Materials)). The predominant compounds identified from MLE were phenols, serving as good sources of substances that mediated the AgNP synthesis. Phenols also serve as precursors for the synthesis of numerous compounds, including flavonoids, phenolic acids, and lignans. They possess various biological and pharmacological properties, including antioxidant activity and the ability to suppress the multiplication of plant-pathogenic bacteria, viruses, and fungi. Due to their high antioxidant activity, polyphenols play an important role in preventing the onset and progression of numerous chronic pathological conditions, such as cancer, diabetes, cardiovascular disease, and aging [16].

Another significant volatile phenolic compound found in the MLE was benzofuranone, a benzofuran derivative identified as one of the significant phytochemical compounds in various *M. alba* extracts. Benzofurans are among the phenolic compounds with many biological activities, including anticancer, antibacterial, antioxidant, and antiviral properties, making them promising natural pharmaceutical compounds [15,17,18].

Other minor phytochemical compounds present in the MLE were benzoyl isothiocyanate and megastigmatrienone. Isothiocyanates (ITCs) are highly reactive organo-sulphur phytochemicals and are the product of the hydrolysis of glucosinolates by the enzyme myrosinase [19,20]. Particularly, aromatic isothiocyanates containing phenethyl-, benzyl-, and benzoyl-groups exhibit a wide range of antimicrobial activity due to their ability to cross and depolarize bacterial membranes [21]. ITCs are also known to possess anticancer activity by inducing apoptosis and cytokine production, as well as anti-inflammatory and cardio-protective properties [22]. It was also found that the MLE contained megastigmatrienone (also known as tabanone), one of the main terpene components. According to a GC–MS study, one of the chemical constituents of mulberry essential oil isolated in Thailand from the silkworm-mulberry leaves, *Morus rotunbiloba* Koidz, is megastigmatrienone. It was found that the essential oil of *M. rotunbiloba* leaf had antibacterial and cytotoxic effects on cancer cell lines [23].

### 2.2. Biological Synthesis and Characterization of MLE-AgNPs

**Figure 1:**
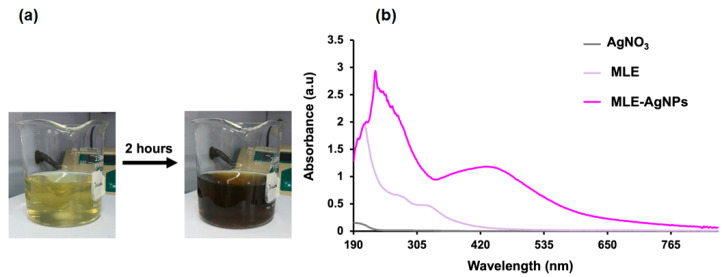
Characterization of AgNPs using white mulberry leaf extract (MLE-AgNPs). Changing color of the reaction mixture after 2 h incubation time (**a**); UV–Vis spectra of MLE-AgNPs compared with either silver nitrate solution (AgNO_3_) or MLE alone (**b**).

The formation of MLE-AgNPs was completed in approximately 2 h (Figure 1a), indicated by the dark brown color in the reaction mixture containing silver nitrate and white mulberry leaf extract. This color change is due to the excitation of the surface plasmon on the metal nanoparticles [24,25]. This was confirmed by observing an intense absorption band at 420 nm (Figure 1b). This band arises from the collective oscillation of surface-plasmon electrons in MLE-AgNPs [26]. This absorption was not observed for either the silver nitrate solution (AgNO_3_) alone or the aqueous extract of white mulberry leaves alone (Figure 1b).

**Figure 2:**
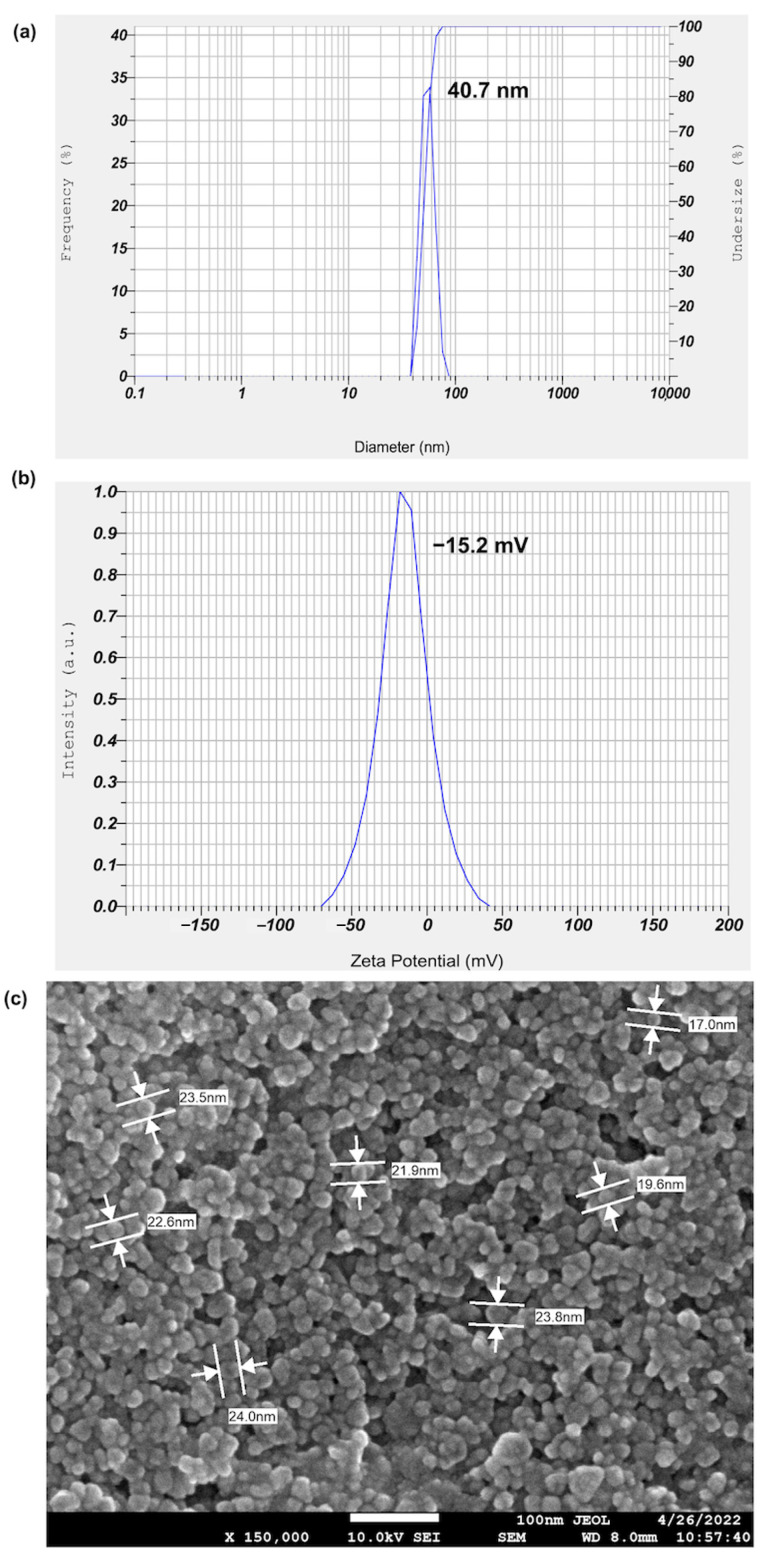

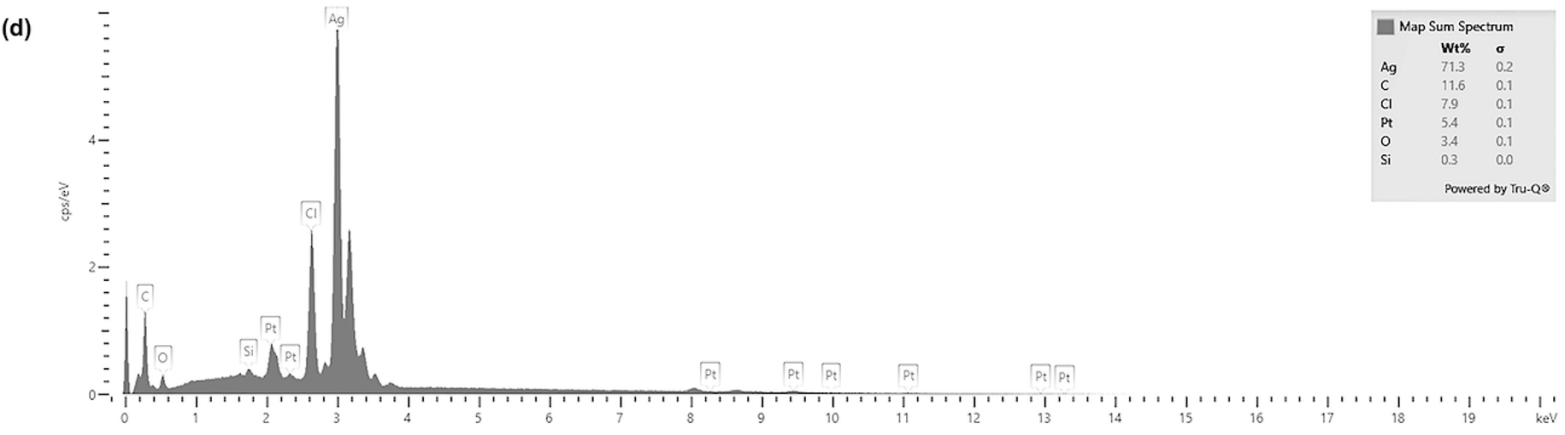
Dynamic light scattering (DLS) (**a**), zeta potential (**b**), FE-SEM image (**c**) and EDX (**d**) analyses of MLE-AgNPs.

Dynamic light scattering (DLS) was used to determine silver nanoparticles’ hydrodynamic size distribution and surface charge. This technique determines the particle’s size from the scattered light caused by the Brownian motion of the particles. The intensity of the scattered light is proportional to the square of the volume of the particle, which makes DLS very sensitive to the presence of large particles [27,28]. The average particle size of biosynthesized MLE-AgNPs determined by dynamic light scattering (DLS) was 44.5 ± 1.19 nm (Figure 2a). The average zeta potential, which is an indicator of surface charge potential and nanoparticles stability in aqueous suspensions, was found to be approximately −14.5 ± 1.79 mV (Figure 2b). The negative potential value could be due to the capping of phenolic constituents present in the MLE, resulting in the repulsion force between adjacent, similarly negatively charged particles that prevent nanoparticle aggregation [26]. It has been reported that AgNPs obtained by the reduction with plant extracts most often are negatively charged [2]. Moreover, highly negatively charged particles tend to repel each other, thus forming stable colloidal solutions which show only minor trends to agglomerate, indicating the stability of AgNP colloid [29].

The elemental composition of MLE-AgNPs was determined by using FE-SEM equipped with an EDX detector. The micrographs obtained (150,000×) showed uniform and spherical shapes of MLE-AgNPs, and their size was in the range of 17.0–24.0 nm (Figure 2c). The particle size observed by FE-SEM was smaller than the average particle size obtained from DLS analysis, likely because the DLS technique measures the diffusion coefficient of suspended nanoparticles undergoing Brownian motion in a solution by analyzing the fluctuating scattered intensity from the nanoparticles in the solution rather than the physical size [30].

The EDX spectrum showed the presence of silver in MLE-AgNPs, with an intense characteristic peak at 3 keV [26,31,32], which confirmed it as the major component (~70%). In addition to the significant peak of Ag, weak signals from C, Cl, Pt, O, and Si were detected, most likely due to phytochemicals capping AgNPs (Figure 2d).

To use nanoparticles as drug delivery systems, they should be in the range of 10–100 nm. Larger particles are captured by macrophages, whereas smaller particles undergo rapid leakage into blood capillaries and renal elimination [31], even though AgNPs smaller than 10 nm can pass through the nuclear pore and interact with chromosomes and DNA, which is advantageous for gene therapy and diagnostics but probably not suitable for drug delivery [33]. For nanomedical applications, the preferred size of nanoparticles is less than 200 nm [34]. In this study, the MLE-AgNPs are spherical with a size of less than 100 nm, suggesting the proper size for drug delivery.

**Figure 3:**
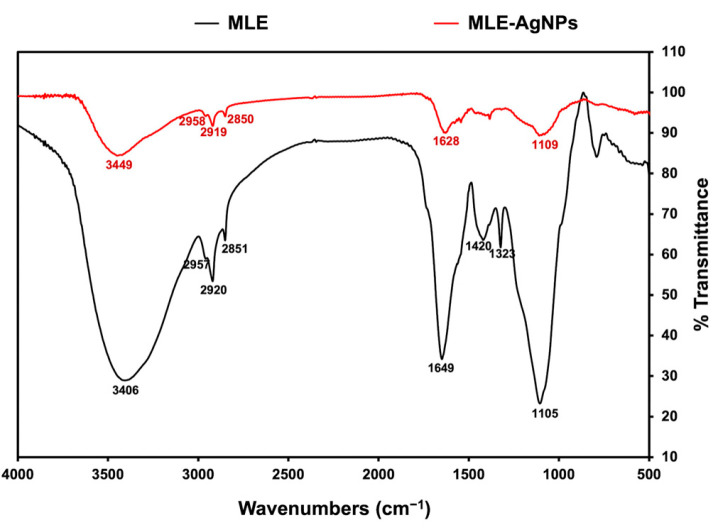
FTIR spectrum of MLE (black) and MLE-AgNPs (red).

FTIR spectra were recorded to investigate the functional groups of MLE-AgNPs compared with those of MLE, as shown in Figure 3. FTIR results demonstrated the transmittance of MLE at 3406, 2957, 2920, 2851, 1649, 1420, 1323, and 1105 cm^−1^, whereas MLE-AgNPs exhibited spectral peaks at 3449, 2958, 2919, 2850, 1628, and 1109 cm^−1^. The peaks at 2957, 2929, and 2851 cm^−1^, representing the C-H stretching, and the peak at 1105 cm^−1^, representing the C-O stretching, remained unchanged in the FTIR spectra of MLE-AgNPs, indicating that these functional groups may not have been reactive during the MLE-AgNPs biosynthesis. The broad peak in the region around 3406 cm^−1^ of MLE is characteristic of the OH stretching commonly found in phenolic compounds, whereas MLE-AgNPs showed a strong shift to the higher frequency at 3449 cm^−1^, indicating that OH groups found in phenolic compounds are responsible for the reduction of silver ions to silver nanoparticles and may interact with AgNPs as a stabilizing or capping agent. In addition, a slight shift of the MLE-AgNPs spectrum appeared in the region at 1628 cm^−1^. This represents the amide I band associated with carbonyl stretching, suggesting that a carbonyl group of amino acid residues present in MLE is involved in the bio-reduction of MLE-AgNPs. The disappearance of the peaks at 1420 and 1323 cm^−1^, which correspond to the O-H bending, in MLE-AgNPs suggests that certain functional groups abundant in MLE, such as OH of phenolic compounds [17], function as reducing and capping agents.

**Figure 4:**
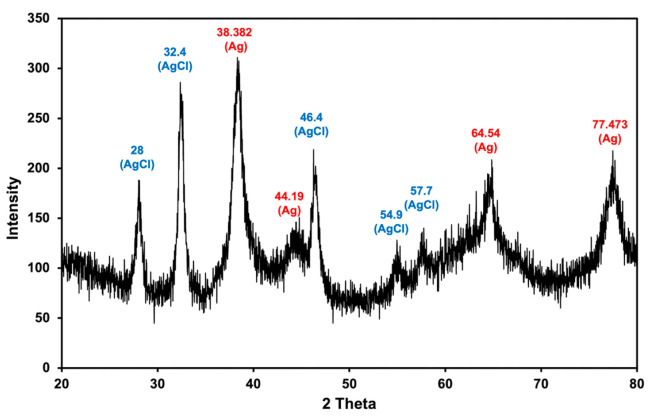
XRD spectra of biosynthesized MLE-AgNPs.

The XRD pattern was obtained to verify the crystalline nature of MLE-AgNPs (Figure 4). Four major XRD diffraction peaks at 2θ values were found at 38.38° (111), 44.19° (200), 64.54° (220), and 77.47° (311), which correspond to the crystallographic planes of face-centered cubic (*fcc*) of silver crystal as reported by JCPDS (Joint Committee on Power Diffraction Standards) card No. 00–004-0783 [35,36,37]. Similar XRD patterns have been demonstrated by other studies using green synthesis methods to formulate AgNPs [35,37,38,39], suggesting the nanocrystalline nature of MLE-mediated AgNPs in this study. Additionally, peaks observed at 20 values of 28°, 32.4°, 46.4° and 57.7° could be indexed to the (111), (200), (311) and (400) planes, respectively, of the AgCl-NPs (JCPDS card No. 31–1238) as previously reported [40]. The formation of AgCl-NPs may result from the reaction between Ag^+^ from AgNO_3_ and Cl^-^ from phytochemical compounds in MLE. Some additional diffraction peaks were probably due to the presence of phytochemical components of the extract.

### 2.3. MLE-AgNPs Exhibited Antibacterial Activity against Gram-Negative and Gram-Positive Bacteria

The antibacterial activity of MLE and MLE-AgNPs against Gram-negative (*Acinetobacter baumannii*, *Escherichia coli* and *Salmonella typhimurium*) and Gram-positive (*Bacillus subtilis* and *Staphylococcus aureus*) bacteria was evaluated using the minimum inhibitory concentration assay (MIC). The MLE-AgNPs were equally effective against Gram-negative and Gram-positive bacteria with a MIC of 32 µg/mL; however, they exhibited superior antibacterial activity against *A. baumannii* strains with a MIC of 2 µg/mL (Table 2). MLE had no bactericidal action at the highest concentration tested (64 µg/mL) (Table 2). This may be explained by the mulberry leaf extract’s poor permeability and low intracellular retention due to its hydrophilic nature. In contrast, the biosynthesized AgNPs mediated by MLE can permeate the thick and rigid cell walls of Gram-positive bacteria and the robust lipopolysaccharide membrane of Gram-negative bacteria due to their small size [41]. The ionization of internalized AgNPs to release Ag^+^ contributes to the formation of intracellular reactive oxygen species (ROS), which ultimately induce cell death by causing damage to proteins, DNA, RNA, lipids, and other essential constituents [42,43].

### 2.4. MLE and MLE-AgNPs Exhibited Anticancer Activity against Breast Cancer Cells

**Figure 5:**
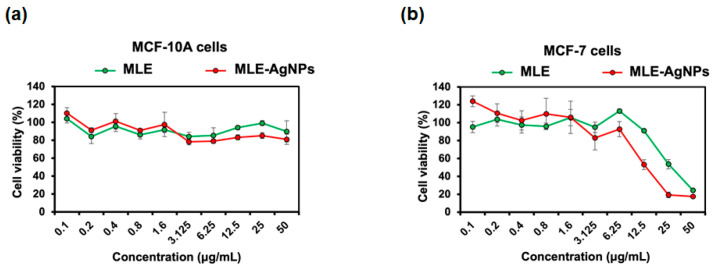
Cell viability of MCF-10A (**a**) and MCF-7 (**b**) cell lines in response to MLE and MLE-AgNPs treatments.

In addition to antibacterial activity, MLE and MLE-AgNPs were also assessed for their anticancer potential. After 48 h of incubation, the MTT assay was used to examine the effect of different concentrations of MLE and MLE-AgNPs on the proliferation of MCF-7 breast cancer cells and MCF-10A normal mammary epithelial cells. The half-maximal inhibitory concentration (IC_50_) for MLE-AgNPs against breast cancer (MCF-7) cells was determined to be 18 µg/mL, while normal MCF-10A cells were unaffected (Figure 5). In addition, MLE inhibited the proliferation of MCF-7 cells by 40–70% at concentrations ranging from 6.25 to 50 µg/mL, giving an IC_50_ value of 33 µg/mL, without affecting the normal MCF-10A cell line (Figure 5). Previous studies have demonstrated the cytotoxic effects of *M. alba* extracts on human colon cancer cells (HCT-15) and breast cancer cells (MCF-7) via apoptosis induction and downregulation of nitric oxide [13,44]. In addition, mulberry leaf lectin (MLL) isolated from *M. alba* leaf extract exhibited antiproliferative activity against MCF-7 breast and HCT-15 colon cancer cells with IC_50_ values of 8.5 and 16.0 µg/mL by caspase-dependent activation of apoptosis [45]. The presence of phenolic compounds such as flavonoids, benzofurans, chalcones, and alkaloids in crude extracts or isolated components of *M. alba* leaves was found to be related to anticancer activity in human cancer cell lines [17]. In this study, the MLE contains phenols and other volatile phenolic compounds, such as benzofuranone, which likely contributed to the anticancer activity of the MLE and MLE-AgNPs. Our results also indicate that the MLE-mediated AgNPs could significantly enhance the cytotoxicity of breast cancer cells compared with the MLE alone. This may be due to the small size and the presence of phenolic compounds as functional groups on MLE-AgNPs. Similar research has found that AgNPs formulated with *M. alba* leaf extract possess greater cytotoxic and hepatoprotective properties than a crude *M. alba* extract, with IC_50_ values of 20 and 80 µg/mL, respectively [46]. It has been shown that AgNPs have elevated cytotoxic activity due to their large surface-to-volume ratio, which allows them to be rapidly ingested into cells and negatively impacts cellular signaling pathways upon interaction with cellular components. Interaction between AgNPs and mitochondria has been reported to disrupt the electron transport chain and increase reactive oxygen species (ROS) levels. Therefore, excessive ROS causes oxidative damage to DNA, proteins, and lipids, ultimately leading to cell death [47,48].

The potential applications of plant-mediated AgNPs in breast cancer treatments have been the subject of extensive research. Plant-mediated AgNPs using aqueous garlic, green tea and turmeric extracts were reported for anticancer activity against human cancer cell lines including breast cancer cells (MCF-7). The IC_50_ values for these plant-mediated AgNPs ranged from 12 to 20 µg/mL, indicating their efficacy in killing MCF-7 cells [49]. In addition, green AgNPs synthesized from *Cynara scolymus* leaf extract in conjunction with photodynamic therapy (PDT) therapy demonstrated potent anticancer activity by inducing mitochondrial apoptosis in MCF-7 cells, with an IC_50_ approximately 10 µg/mL [24]. Another study showed that the biosynthesized AgNPs using *Mentha arvensis* leaf extract induced more than 50% cell death when treating the MCF-7 cells at 6.25 μg/mL [50]. Similarly, AgNPs synthesized by using *Dendrophthoe falcata* leaf extract exhibited cytotoxicity against MCF-7 cells in a dose-dependent manner, giving an IC_50_ value of 5 μg/mL [51].

## 3. Materials and Methods

### 3.1. Bacteria Strains

Gram-negative bacteria including *Acinetobacter baumannii* (ATCC 17978 and ATCC 19606), *Escherichia coli* (ATCC 25922), *Salmonella typhimurium* (DMST 562), Gram-positive bacteria including *Bacillus subtilis* (PY79), and *Staphylococcus aureus* (ATCC 29213) were used in this study.

### 3.2. Collection and Preparation of Aqueous Extract of Morus alba Leaves

Fresh leaves of white mulberry (*Morus alba* L.) were collected from a local area in Samut Sakhon, Thailand and dirt was removed by washing with sterile water. The washed leaves were sun-dried to remove residual moisture and were cut into small pieces. Finely chopped leaves (5 g) were added to 100 mL of boiled sterile water and stirred at 75 °C for 2 h. After boiling, the mixture was cooled at room temperature and filtered through cheesecloth. The aqueous extract of *M. alba* leaves (MLE) was stored at 4 °C for further use.

### 3.3. Gas Chromatography and Mass Spectrometry (GC–MS) Analysis

GC–MS analysis of MLE was used to determine its phytochemical composition. The GC–MS analysis was conducted at the Mahidol University-Frontier Research Facility (MU-FRF), Thailand using a GC–MS 7890A-5977B (Agilent Technologies, Santa Clara, CA, USA). The HP-5MS (5%-phenyl)-methylpolysiloxane column was attached to the GC–MS ultra-system. The mixture was filtered through a 0.45 μm syringe. The MLE (2 mL) was placed in a 20-mL magnetic-top-sealed vial. Each vial was heated at 70 °C for 30 min to reach sample headspace equilibrium. The volatile compounds were extracted using a 50/30 µm divinylbenzene–carboxen-polydimethylsiloxane (DVB/CAR/PDMS) fiber (Supelco, Merck KGaA, Bellefonte, PA, USA). The fiber was inserted into the vial and exposed to the headspace above the MLE sample for 20 min at 70 °C. After the extraction, the fiber was thermally desorbed into the GC injection port for 5 min at 300 °C. The inert gas helium (Ultra High Purity, 99.999%) was used as a carrier gas at the constant flow rate of 1.0 mL/min in splitless mode. The column oven temperature was held at 50 °C for 1 min, then increased from 50 to 290 °C at a constant rate of 4 °C/min with a hold time of 2 min, and then increased to 300 °C at a constant rate of 50 °C/min. The beginning *m/z* was 50, and the ending *m/z* was 500. The total run time was 63.2 min. The molecular fragmentation was obtained by electron ionization (EI). The data were obtained in full scan mode, and the mass/charge ratio (*m/z*) was recorded between 50 and 550 at 70 eV. The phytochemical compounds from the GC–MS analyses were identified and presented with their compound names, retention times (RT), and molecular formulas using the database of the National Institute of Standards and Technology (NIST17, Gaithersburg, MD, USA).

### 3.4. Green Synthesis of Silver Nanoparticles (MLE-AgNPs)

A volume of 5 mL of mulberry leaf extract (MLE) was added dropwise to 100 mL of a 1 mM AgNO_3_ solution while stirring at room temperature for approximately 2 h until the solution became colloidal dark brown, indicating AgNP formation. The mixture was centrifuged at 10,000 rpm for 15 min. The pellet containing AgNPs was washed with ultrapure water to remove silver ions and leaf extract residues before use.

### 3.5. Characterization of MLE-AgNPs

#### 3.5.1. UV–Visible Absorbance Spectroscopy

Formation of MLE-AgNPs was observed by using UV–Vis spectroscopy (NanoDrop™ One Microvolume UV–Vis Spectrophotometer, Thermo Fisher Scientific, Waltham, MA, USA) with a wavelength range of 190–850 nm. The reduction of silver ion (Ag^+^) in solution to metallic silver (Ag^0^) nanoparticles was monitored with the strong surface plasmon resonance (SPR) absorption band at 420 nm corresponding to silver colloids.

#### 3.5.2. Dynamic Light Scattering (DLS)

The size and surface charge analysis of the MLE-AgNPs were carried out using dynamic light scattering (Horiba SZ-100 particle size analyzer, Kyoto, Japan). To determine their size distribution and surface charge (Zeta-potential value), MLE-AgNPs were injected into a disposable cell and a zeta-potential cell with carbon-coated electrodes, respectively.

#### 3.5.3. Field Emission Scanning Electron Microscopy (FE-SEM) Coupled with Energy Dispersive X-ray Spectroscopy (EDX), Fourier-Transform Infrared Spectroscopy (FTIR), and X-ray Diffraction (XRD) Analyses

The size, shape, and basic elements on the surface morphology of MLE-AgNPs were characterized by using FE-SEM equipped with EDX(JSM-7610FPlus, JEOL, Tokyo, Japan). The sample mixture was centrifuged at 10,000 rpm at room temperature for 15 min. The pellet was collected and washed several times with ultrapure water, then the pellet was air-dried for 48 h. Prior to FE-SEM and EDX analyses, the thin films of the sample were prepared on a carbon-coated copper grid, and the grid was then coated with platinum to increase conductivity. The functional groups of biosynthesized AgNPs were monitored with FTIR spectroscopy (Nicolet iS50, Thermo Fisher Scientific Co., Waltham, MA, USA). The pellet sample was prepared by the standard KBr pellet method to perform FTIR, and analyzed in the range of 500–4000 cm^−1^ using the transmittance mode. Crystalline metallic AgNPs were also analyzed by using XRD (Bruker D2 Phaser, Bruker AXS GmbH, Karlsruhe, Germany). The 2Theta was measured from 5° to 90° with a step size of 0.02° using Cu/Kα radiation (λ = 1.54184 Å).

### 3.6. Determination of the Minimum Inhibitory Concentration (MIC) of MLE and MLE-AgNPs

The minimum inhibitory concentrations (MICs) were determined using the microdilution method as previously described [52]. Briefly, the well-separated colonies of *A. baumannii*, *E. coli*, *S. typhimurium*, *B. subtilis*, and *S. aureus* were inoculated in tryptic soy broth (TSB) and incubated at 30–32 °C on a roller overnight. Overnight bacteria cultures were then diluted in TSB to 2 × 10^8^ CFU/mL. Two-fold serial dilutions of the MLE (64 µg/mL to 0.125 µg/mL) and MLE-AgNPs (512 µg/mL to 2 µg/mL). The final concentration of bacteria was adjusted to 2 × 10^5^ CFU/mL by diluting the 2 × 10^8^ CFU/mL culture in TSB and adding it to each well of the 96-well plate containing various concentrations of MLE or MLE-AgNPs. Cultures were allowed to grow at 30 °C for 24 h. MICs were determined by observing the concentration at which the bacteria could not grow in the well. Each antibacterial test included one negative growth control containing only TSB media and one positive growth control containing 2 × 10^5^ CFU/mL of bacteria in TSB. At least three independent experiments were conducted for each antibacterial test, and the MIC value was derived from the consensus of the mode values.

### 3.7. In Vitro Cytotoxicity Assay

#### 3.7.1. Cell Lines and Culture Conditions

The human breast adenocarcinoma (MCF-7) and human epithelial cell lines (MCF-10A) were purchased from ATCC (American Type Culture Collection, Manassas, VA, USA). MCF-7 cells were cultured in Eagle’s minimum essential medium (EMEM) (ATCC, Manassas, VA, USA) supplemented with 10% (*v*/*v*) heat-inactivated fetal bovine serum (FBS) (Life Technologies, Paisley, UK) and 1× penicillin/streptomycin (Life Technologies, Grand Island, NY, USA) at 37 °C in an incubator containing 5% CO_2_ and the cells were sub-cultured at regular intervals.

MCF-10A cells were cultured in mammary epithelial cell basal medium (MEBM) (Lonza, Walkersville, MD, USA) supplemented with 0.25× mammary epithelial growth supplement (MEGS) (Life Technologies, Grand Island, NY, USA), 5% (*v*/*v*) horse serum (Biological Industries, Beit-Haemek, Israel) and 1× penicillin/streptomycin (Life Technologies, Grand Island, NY, USA) at 37 °C in an incubator containing 5% CO_2_ and the cells were sub-cultured at regular intervals.

#### 3.7.2. Antiproliferative Analysis

Cell viability was assessed using an MTT (3-(4,5-dimethylthiazol-2-yl)-2,5-diphenyltetrazolium bromide) assay. Cells treated with MLE-AgNPs were compared to cells treated with MLE. Briefly, 1 × 10^4^ cells were seeded into each well of a 96-well plate and cultured for 48 h. MLE-AgNPs or MLE was added to the wells at concentrations ranging from 0.1 to 50 µg/mL (three replicates for each concentration), and the plate was incubated in a CO_2_ incubator at 37 °C for 48 h. Cell morphological alterations in both types of cells were observed using an inverted light microscope (Nikon Eclipse TS100, Melville, NY, USA). MTT (Invitrogen, Carlsbad, CA, USA) solution (10 μL) was added to each well to a final concentration of 5 mg/mL, and the plate was incubated for 4 h. Then, dimethyl sulfoxide (DMSO) (Merck, Darmstadt, Germany) (100 μL) was added to each well to dissolve the formazan crystals, followed by absorbance measurement at 595 nm (Beckman Coulter DTX 880 Microplate Reader). The IC_50_ value was calculated as the test sample concentration required for 50% cell growth inhibition.

### 3.8. Statistical Analysis

The data are presented as the mean ± standard deviation (SD).

## 4. Conclusions

The biosynthesized AgNPs were successfully fabricated using the white mulberry leaf extract (MLE) as reducing, capping, and stabilizing agents. The presence of phenolic compounds analyzed by GC–MS confirmed the therapeutic value of mulberry leaf extract. MLE-AgNPs demonstrated strong antibacterial activity against *A. baumannii* strains and good activity against Gram-negative and Gram-positive bacteria. MLE and MLE-AgNPs showed specific anticancer activity against breast cancer cells, indicating their high potential for breast cancer treatment. Thus, it can be concluded that the compounds of *M. alba* leaves, serving as reducing and capping agents for AgNPs biosynthesis, possess a therapeutic potential and enhance the efficacy of biosynthesized AgNPs.

## Figures and Tables

**Table 1:** Bioactive compounds of *M. alba* leaf extract (MLE) analyzed by GC–MS.

S. No.	RT (min)	Compound Name	Formula
1	6.953	Benzoyl isothiocyanate	C_8_H_5_NOS
2	25.080	Phenol, 3,5-bis(1,1-dimethylethyl)	C_14_H_22_O
3	25.554	2(4*H*)-Benzofuranone, 5,6,7,7*a* tetrahydro-4,4,7*a*-trimethyl	C_11_H_16_O_2_
4	27.038	Megastigmatrienone	C_13_H_18_O

**Table 2:** Minimum inhibitory concentration (MIC) of MLE and MLE-AgNPs against Gram-negative and Gram-positive bacteria.

Bacterial Strain		MIC (µg/mL)	
		MLE	MLE-AgNPs
Gram-negative	*Acinetobacter baumannii* (ATCC 17978)	>64	2
	*Acinetobacter baumannii* (ATCC 19606)	>64	2
	*Escherichia coli* (ATCC 25922)	>64	32
	*Salmonella typhimurium* (DMST 562)	>64	32
Gram-positive	*Bacillus subtilis* (PY59)	>64	32
	*Staphylococcus aureus* (ATCC 29213)	>64	32

## Data Availability

All the data have been presented in this article.

## Supplementary Materials

The following supporting information can be downloaded at: www.mdpi.com/article/10.3390/molecules28031213/s1, Figure S1: Gas chromatography and mass spectrometry (GC–MS) analysis of an aqueous Morus alba leaf extract (MLE).

## Sample Availability

Samples of the compounds are available from the authors.
