# Supplementary material for: Biosynthesized Silver Nanoparticles Using Morus alba (White Mulberry) Leaf Extract as Potential Antibacterial and Anticancer Agents
Source: Molecules. 2023 Jan 26;28(3):1213. doi: 10.3390/molecules28031213 (PMC9920803; doi:10.3390/molecules28031213)
Supplement: Supplementary file 1 [file molecules-28-01213-s001.zip › molecules-2192233-supplementary.pdf]

Supplementary Materials

Biosynthesized Silver Nanoparticles Using *Morus alba* (White Mulberry) Leaf Extract as Potential Antibacterial and Anticancer Agents

Tipaporn Kumkoon, Monrudee Srisaisap and Panadda Boonserm *

Institute of Molecular Biosciences, Mahidol University, Nakhon Pathom 73170, Thailand; tipapornkumkoon@gmail.com (T.K.); catta_w255@hotmail.co.th (M.S.)

* Correspondence: panadda.boo@mahidol.ac.th

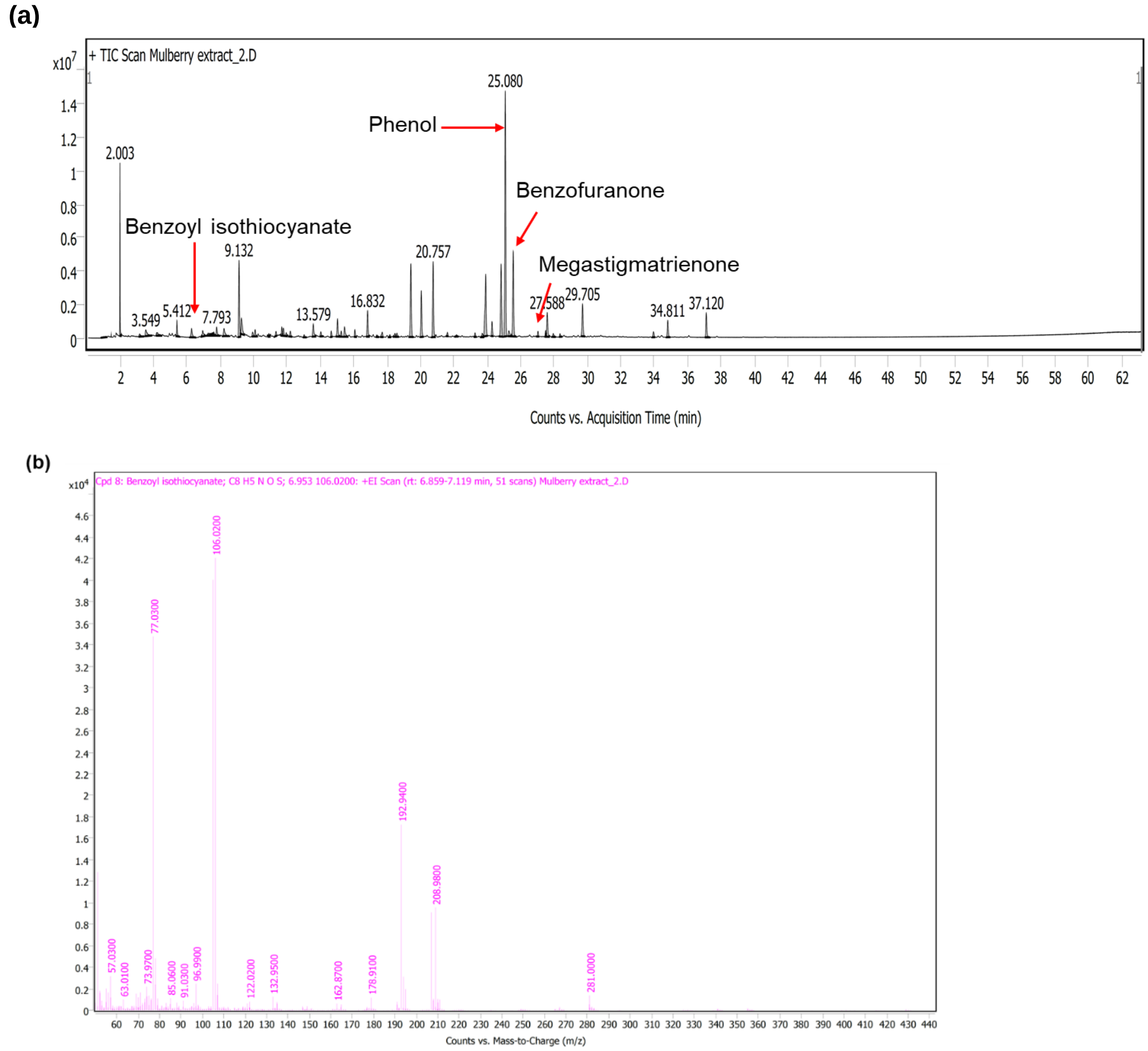

(c)

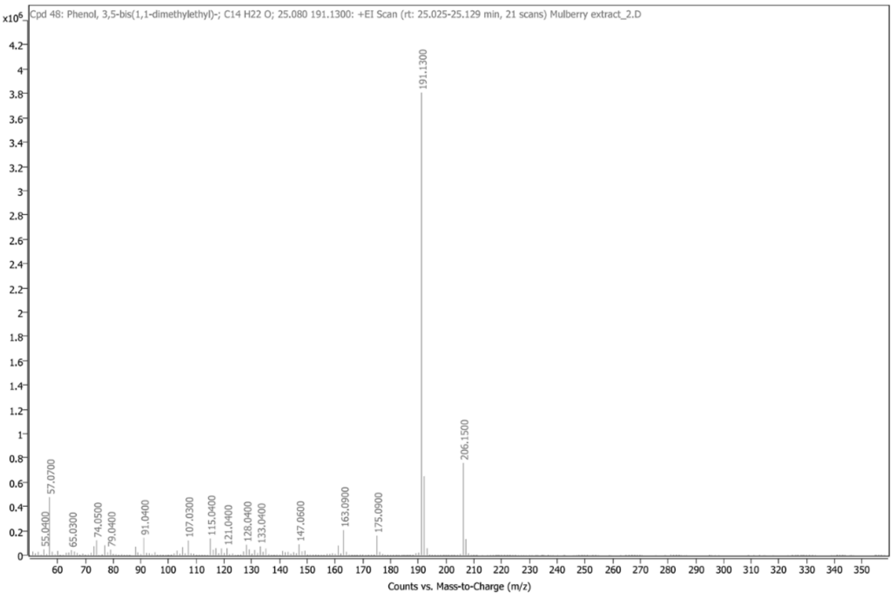
x10^6
Cpd 48: Phenol, 3,5-bis(1,1-dimethylethyl)-; C14 H22 O; 25.080 191.1300: +EI Scan (rt: 25.025-25.129 min, 21 scans) Mulberry extract_2.D
191.1300
206.1500
57.0700
55.0400
65.0300
74.0500
79.0400
91.0400
107.0300
115.0400
121.0400
128.0400
133.0400
147.0600
163.0900
175.0900
Counts vs. Mass-to-Charge (m/z)

(d)

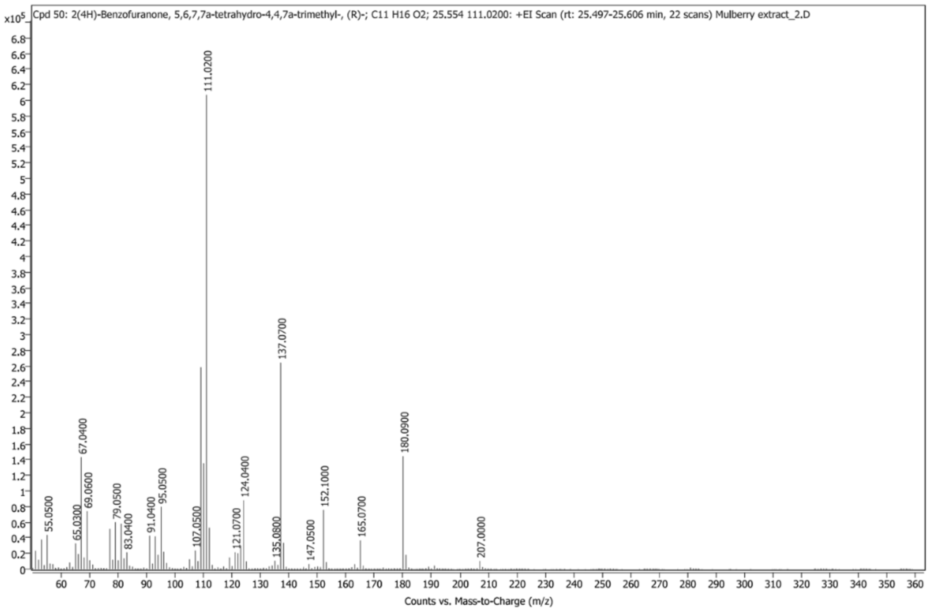
x10^5
Cpd 50: 2(4H)-Benzofuranone, 5,6,7,7a-tetrahydro-4,4,7a-trimethyl-, (R)-; C11 H16 O2; 25.554 111.0200: +EI Scan (rt: 25.497-25.606 min, 22 scans) Mulberry extract_2.D
111.0200
137.0700
180.0900
67.0400
124.0400
55.0500
65.0300
69.0600
79.0500
83.0400
91.0400
95.0500
107.0500
121.0700
135.0800
147.0500
152.1000
165.0700
207.0000
Counts vs. Mass-to-Charge (m/z)

(e)

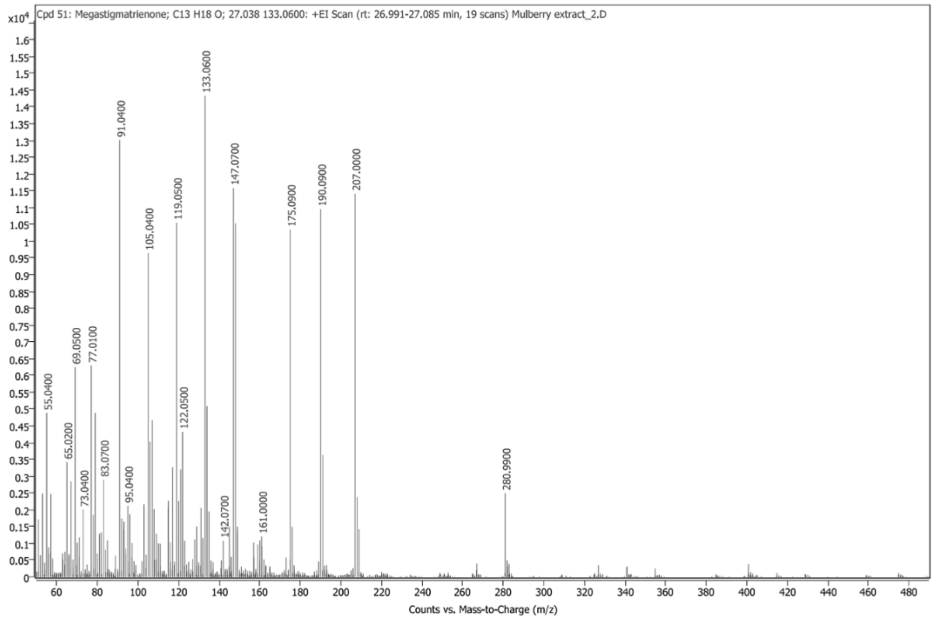

Figure S1. Gas chromatography and mass spectrometry (GC-MS) analysis of an aqueous *Morus alba* leaf extract (MLE). GC-MS chromatogram of MLE showing peaks of significant bioactive compounds, including phenol, benzofuranone, benzoyl isothiocyanate, and megastigmatrienone (a). The mass spectra are shown for benzoyl isothiocyanate (b), phenol (c), benzofuranone (d), and megastigmatrienone (e).
